# Advances in Stereotactic Body Radiation Therapy: Expanding Clinical Applications and Improving Treatment Precision

**DOI:** 10.3390/cancers18203402

**Published:** 2026-10-21

**Authors:** Michael D. Chuong, Sam Beddar

**Affiliations:** 1 Department of Radiation Oncology, Herbert Wertheim Cancer Institute, Baptist Health South Florida, Miami, FL 33176, USA; 2 Department of Oncological Sciences, Florida International University Herbert Wertheim College of Medicine, Miami, FL 33199, USA; 3 Department of Radiation Physics, Division of Radiation Oncology, The University of Texas MD Anderson Cancer Center, Houston, TX 77030, USA; 4 The University of Texas MD Anderson Cancer Center UTHealth Houston Graduate School of Biomedical Sciences, Houston, TX 77030, USA

## 1. Introduction

Stereotactic body radiation therapy (SBRT), also referred to as stereotactic ablative radiotherapy (SABR), was pioneered at the Karolinska Institute in Sweden in the early 1990s, extending the principles of intracranial stereotactic radiosurgery (SRS) to extracranial tumors [1]. Early investigators recognized the importance of accurate target localization, patient immobilization, and respiratory motion management to safely deliver high doses of radiation in a limited number of fractions. Although early studies demonstrated promising local control (LC), reports of severe toxicity, particularly for central lung tumors, emphasized the importance of patient selection, appropriate dose and fractionation, and normal tissue protection [2].

Over the following decades, advances in radiation treatment technology have substantially expanded the clinical applications of SBRT. Improvements in image guidance, treatment planning, motion management, and radiation delivery have enabled the treatment of tumors previously considered unsuitable for ablative radiation. For example, magnetic resonance (MR)-guided radiation therapy (RT) and online adaptive treatment have provided opportunities to account for daily anatomic changes and improve the therapeutic ratio, especially for tumors near radiosensitive organs. These advances are particularly relevant for pancreatic cancer, where conventional radiation doses have historically provided limited durable LC [3]. Furthermore, the ability to safely deliver ablative radiation across a broader range of disease sites has helped foster the integration of SBRT with systemic therapies, including immunotherapy, in both localized and metastatic disease.

This Special Issue of *Cancers*, “Stereotactic Body Radiation and Stereotactic Ablative Radiotherapy Therapy for Cancers, 2nd Edition,” includes studies examining expanding clinical applications of SBRT, advances in image guidance and motion management, and challenges associated with implementing increasingly sophisticated radiation techniques.

## 2. Expanding Clinical Applications of SBRT

Advances in radiation delivery have expanded the clinical applications of SBRT, creating opportunities to integrate ablative radiation into multimodality treatment strategies and offer effective local therapy for patients with limited treatment options. Three studies in this Special Issue illustrate these evolving applications.

In their study, “Stereotactic Body Radiotherapy for Oligometastatic Disease in Melanoma Patients Receiving Immunotherapy: A Single-Center Retrospective Analysis”, Lekić et al. evaluated SBRT in 63 patients with oligometastatic melanoma receiving immune checkpoint inhibitors; they reported a 3-year LC rate of approximately 90% with limited toxicity. The increasing effectiveness of immunotherapy has paved the way for incorporating metastasis-directed SBRT into the management of oligometastatic and oligoprogressive disease, potentially allowing patients to continue otherwise effective systemic therapy. Improvements in image guidance and radiation delivery have facilitated the treatment of extracranial metastases across multiple anatomic sites. Although the authors found no statistically significant differences in outcomes according to the sequencing of SBRT and immunotherapy, their findings add to the evidence supporting the feasibility of this combined approach. Whether SBRT enhances systemic antitumor immunity beyond its local effects remains uncertain.

In a study titled “External Beam Radiation Therapy for Pheochromocytoma and Non-Head and Neck Paraganglioma: A Single-Institution Experience and Systematic Review”, Jin et al. investigated RT for pheochromocytoma and non-head and neck paraganglioma, rare tumors for which surgery is generally preferred when feasible. Although RT has an established role in head and neck paragangliomas, evidence supporting its use at other anatomic sites is more limited. The authors reported a 5-year LC rate exceeding 95% for non-head and neck lesions, with frequent symptomatic improvement and limited severe toxicity. These findings support highly conformal RT as an alternative to surgery for selected patients with tumors in anatomically challenging locations. However, as both stereotactic and conventionally fractionated approaches were included, the study supports RT more broadly rather than establishing a specific advantage of SBRT.

In their paper, “Neoadjuvant Chemotherapy and Stereotactic Body Radiation Therapy in Patients with Early-Onset Pancreatic Cancer: Clinical Outcomes and Toxicity”, Sehgal et al. examined SBRT as part of multimodality treatment for early-onset pancreatic cancer, which is an understudied population. Among 47 patients younger than 55 years treated with induction chemotherapy followed by SBRT, longer chemotherapy duration and CA 19-9 normalization were associated with improved outcomes, although these associations may be influenced by patient selection and treatment-related timing biases. Despite multimodality treatment, 1-year local progression-free survival was only 45%, highlighting the challenge of achieving durable disease control. Nearly all patients received 33 Gy in five fractions, which is not an ablative regimen. These findings raise the question of whether radiation dose escalation could improve outcomes in appropriately selected patients, although one patient experienced fatal radiation-related gastrointestinal bleeding. Advances in image guidance, motion management, and online adaptive RT may facilitate safer delivery of ablative doses near radiosensitive gastrointestinal organs, potentially addressing a longstanding limitation of pancreatic SBRT [3].

## 3. Advances in Image Guidance, Motion Management, and Treatment Precision

Safe delivery of ablative radiation depends on accurate target localization and limiting radiation exposure to surrounding normal tissues. Despite improvements in image guidance and treatment delivery, inter- and intrafraction anatomic changes remain important challenges. Five studies in this Special Issue examine approaches to improving treatment accuracy, efficiency, and clinical implementation.

Kaiser et al.’s study, “Ablative Five-Fraction CT Versus MR-Guided Stereotactic Body Radiation Therapy for Pancreatic Cancer: In Silico Evaluation of Interfraction Anatomic Changes as a Rationale for Online Adaptive Replanning”, evaluated the dosimetric consequences of interfraction anatomic changes during ablative pancreatic SBRT prescribed to 50 Gy in five fractions. This in silico comparison of nonadaptive CT-guided SBRT and MR-guided online adaptive treatment demonstrated that 96% of fractions would have exceeded at least one gastrointestinal organ-at-risk (OAR) constraint without adaptation. Online adaptive replanning allowed gastrointestinal constraints to be satisfied while maintaining target coverage. These findings support the use of online adaptation to safely deliver ablative pancreatic SBRT.

In their study, “Optimizing Motion Management and Baseline Shifts in Magnetic Resonance-Guided Spine Stereotactic Body Radiation Therapy”, Ding et al. investigated intrafraction motion management during MR-guided spine SBRT, where small positional changes can substantially affect spinal cord dose. Using real-time cine MRI, the authors evaluated intrafraction motion and simulated baseline position corrections. Although baseline shifts improved simulated treatment delivery efficiency, they also increased spinal cord dose, exceeding the specified constraint in three out of five cases. These findings illustrate how interventions intended to improve treatment efficiency may introduce dosimetric risks, emphasizing the importance of evaluating the consequences of motion management strategies before clinical implementation.

Sadagopan et al. evaluated the effects of CT acquisition speed on localization accuracy during thoracic SBRT in a study titled “Reduced Computed Tomography Scan Speed Improves Alignment Errors for Patients Undergoing Thoracic Stereotactic Body Radiation Therapy”. Using a respiratory motion phantom, the authors demonstrated that slower CT acquisition reduced alignment errors compared with conventional acquisition protocols. This study illustrates how modifications to existing image guidance techniques may improve treatment accuracy without requiring new radiation delivery platforms.

“Longitudinal Improvements in Treatment Delivery Efficiency for MR-Guided Radiation Therapy: An 8-Year Single-Institution Experience” is a retrospective analysis by Herrera and colleagues, who reported institutional changes in treatment delivery efficiency with MR-guided adaptive RT. In an 8-year institutional experience encompassing 1026 patients and 7665 fractions, median in-room time for online adaptive treatment decreased from 67 to 48 min despite increasing treatment complexity and utilization of adaptation. Nearly 80% of adaptive fractions were completed within 60 min during the later study period. These findings demonstrate that technological improvements, workflow optimization, and institutional experience can enable more efficient delivery of complex treatments, potentially reducing a barrier to the broader adoption of MR-guided adaptive RT. However, the single-institution experience may not be directly generalizable to other treatment platforms or institutions.

Mackin et al. addressed the complementary challenge of safely implementing spine stereotactic RT in clinical practice in their study, “Clinical Workflow of Spine Stereotactic Radiotherapy and Radiosurgery: Insights from a Single-Institution Physics Perspective”. Their technical report describes a multidisciplinary workflow encompassing patient selection, immobilization, multimodality imaging, treatment planning, quality assurance, and image-guided delivery. Of particular interest is the emphasis on structured training and credentialing for physicians and physicists. Standardized workflows, training, and multidisciplinary expertise are essential for the safe implementation of spine SBRT.

These studies collectively demonstrate how improvements in image guidance, motion management, adaptive treatment planning, and treatment efficiency are addressing technical limitations of SBRT. They also emphasize the need to critically evaluate new approaches, particularly when improvements in one aspect of treatment delivery may introduce additional risks or complexity.

## 4. Future Directions and Conclusions

Looking ahead, an important priority will be determining whether the dosimetric advantages of emerging technologies translate into improvements in tumor control, toxicity, and quality of life. Although greater treatment precision may facilitate dose escalation and reduce normal tissue exposure, prospective evidence demonstrating improved clinical outcomes remains limited for many emerging applications.

Another important direction is the integration of SBRT with increasingly effective systemic therapies. Advances in immunotherapy and targeted treatments have created opportunities to use SBRT for oligometastatic and oligoprogressive disease, potentially prolonging the benefit of systemic treatment. Improved systemic disease control may allow more patients with aggressive malignancies, such as pancreatic cancer, to benefit from durable LC. Optimizing patient selection, radiation dose and fractionation, and treatment sequencing will be essential.

Continued technological innovation, including online adaptive RT, automated treatment planning, and artificial intelligence-assisted workflows, may further improve the precision and efficiency of SBRT. However, broader adoption will require consideration of clinical benefit, treatment complexity, resource requirements, and reproducibility across institutions. The studies in this Special Issue illustrate that the continued evolution of SBRT depends on advances in clinical application, treatment precision, and implementation.
